# A curated arterial stiffness dataset for vascular age prediction in China

**DOI:** 10.1038/s41597-026-07276-2

**Published:** 2026-04-28

**Authors:** Xiaohui Chen, Pengcheng Ding, Mengbo He, Sai Zhang, Qingfeng Tang, Guangjun Wang, Zhifeng Tian, Hui An

**Affiliations:** 1 School of Information Technology, Anqing Vocational and Technical College, Anqing, 246133 China; 2 Digital and Intelligent Health Research Center, Anqing Normal University, Anqing, 246133 China; 3 The University Key Laboratory of Intelligent Perception and Computing of Anhui Province, Anqing Normal University, Anqing, 246133 China; 4 Health Management & Physical Examination Center, Xiangyang Central Hospital, Affiliated Hospital of Hubei University of Arts and Science, Xiangyang, 441021 China

**Keywords:** Ageing, Arterial stiffening

## Abstract

Arterial stiffness is an important biomarker of cardiovascular health, and vascular age (VA) prediction provides additional value beyond chronological age. Here we present a curated arterial stiffness dataset comprising 36,223 participants aged 30–80 years from China. To benchmark its utility for VA modelling, we evaluated the Klemera–Doubal Method (KDM) and six Artificial Intelligence (AI) models: multiple linear regression, LASSO, random forest, support vector regression, XGBoost, and a deep neural network. Results showed that the dataset enables VA prediction using both statistical and learning-based approaches. Across both male and female cohorts, KDM showed the lowest prediction error under the current benchmark setting, while several nonlinear learning-based models achieved better performance than the linear baselines. Among the learning-based methods evaluated here, SVR and XGBoost showed comparatively strong performance. This dataset provides a useful open resource for vascular aging research, cardiovascular risk assessment, and methodological benchmarking.

## Background and Summary

Atherosclerosis is a chronic and progressive vascular disease characterized by endothelial dysfunction, lipid deposition, inflammatory responses, and eventual plaque formation within the arterial wall^1^. Over time, these pathological processes result in arterial wall thickening, lumen narrowing, and a significant loss of arterial elasticity. Arterial stiffness, one of the most prominent manifestations of atherosclerosis^2^, alters the hemodynamic environment by reducing the buffering capacity of arteries and increasing both systolic blood pressure and pulse pressure^3^. These changes impose a higher workload on the heart, accelerate left ventricular remodelling, and contribute to end-organ damage in the brain and kidneys. Numerous epidemiological studies have established arterial stiffness as a strong, independent predictor of cardiovascular events, including myocardial infarction and stroke^4^. Therefore, arterial stiffness assessment not only reflects the degree of vascular damage but also serves as an important biomarker for the early detection and risk stratification of cardiovascular disease.

Vascular age (VA) has emerged as a concept to better capture the biological state of the arterial system compared with chronological age (CA)^5^. CA alone fails to account for interindividual variability in vascular health, whereas VA incorporates structural and functional markers that directly reflect arterial condition. VA prediction models integrate demographic variables, clinical risk factors, and vascular function indicators such as pulse wave velocity, ankle-brachial index, and central blood pressure^6,7^. These models allow for an individualized assessment of vascular health and can identify participants who experience “premature vascular aging,” despite being younger in chronological terms^8^. Compared with conventional cardiovascular risk scores, VA prediction provides patients with more intuitive feedback and has been shown to improve awareness and adherence to preventive strategies^9^. Furthermore, with advances in machine learning and artificial intelligence (AI), increasingly accurate models are being developed to estimate VA^10^, enabling a shift toward precision cardiovascular medicine^11^.

Atherosclerosis is intrinsically linked to vascular aging, making it a central factor in VA prediction. Structural and functional alterations caused by atherosclerosis, including arterial stiffening, impaired vasodilation, and increased pulse wave velocity, directly accelerate vascular aging processes^12^. Clinical studies have consistently demonstrated that individuals with higher arterial stiffness exhibit older VA relative to their CA, even after adjusting for traditional risk factors^13^. Incorporating atherosclerosis-related markers into predictive models significantly enhances their predictive power and clinical utility^14^. For example, pulse wave velocity and ankle-brachial index–both surrogate markers of arterial stiffness–are widely recognized as independent predictors of cardiovascular events and are integral to VA algorithms^15^. Thus, atherosclerosis not only underpins the pathophysiological basis of vascular aging but also provides quantifiable, noninvasive biomarkers that can be effectively utilized in predictive models. This dual role underscores the value of incorporating atherosclerosis-related measures into VA prediction frameworks to improve predictive accuracy and clinical relevance^16^.

To address these challenges, the present study aims to establish a comprehensive dataset derived from atherosclerosis-related measurements, specifically designed to support the development and validation of VA prediction models. By curating multi-dimensional data reflecting arterial structure and function, this work seeks to provide an open-access resource for the research community. Such a dataset will not only facilitate benchmarking of conventional statistical methods and advanced machine learning algorithms, but also enable systematic evaluation of deep learning models, thereby accelerating methodological innovation. Ultimately, this open resource is intended to facilitate reproducible research on vascular aging, support benchmarking of predictive methods, and promote broader clinical and translational studies in cardiovascular health.

## Methods

### Ethical approval

The present study utilized de-identified medical record data in a retrospective manner, ensuring that no patient identity or further clinical procedures were involved. Following the guidelines of the Declaration of Helsinki and the exemption policy of the Ethics Committee at Anqing Normal University, ethical review approval was waived (Approval No. AQNU2025039).

### Data acquisition

The dataset contains arterial stiffness data from 36,223 participants (22,959 males and 13,264 females), with the detailed characteristics of the study population summarized in Table 1. These data were obtained through a research program conducted at Xiangyang Central Hospital between January 2015 and August 2022, involving individuals undergoing routine health examinations. The dataset comprises two major components: (1) comprehensive measurements of arterial stiffness parameters, and (2) key demographic information, including age and sex.

**Table 1:** Statistics of the arterial stiffness dataset.

Feature Name	Summary	Male	Female	*P* value
Participants (n)	36223	22,959	13,264	—
Age (years)	50.86±11.96	51.03±12.08	50.57±11.75	<0.001
LbaPWV(m/s)	14.66±3.32	14.98±3.33	14.11±3.23	<0.001
RbaPWV(m/s)	14.64±3.16	14.98±3.12	14.05±3.15	<0.001
LbPMap(mmHg)	53.85±3.20	53.67±3.25	54.16±3.10	<0.001
RbPMap(mmHg)	53.87±3.18	53.64±3.26	54.26±3.06	<0.001
LaSYS(mmHg)	144.29±25.53	148.25±24.58	137.43±25.69	<0.001
RaSYS(mmHg)	145.02±25.42	148.87±24.43	138.35±25.71	<0.001
LaMAP(mmHg)	99.83±15.54	102.15±15.04	95.81±15.56	<0.001
RaMAP(mmHg)	100.01±15.56	102.34±15.09	95.98±15.54	<0.001
Labi	1.12±0.10	1.13±0.09	1.10±0.10	<0.001
Rabi	1.12±0.09	1.13±0.09	1.11±0.09	<0.001

Continuous variables are presented as mean±standard deviation (SD). Group comparisons between males and females were performed using Welch’s two-sample t-test, which does not assume equal variances.

To minimize batch effects, a standardized data acquisition protocol was implemented. First, this study utilized the BP-203RPE III device (Omron, Japan)^17^, an arterial stiffness assessment instrument equipped with pressure sensors capable of simultaneously recording the ankle-brachial index (ABI) and brachial-ankle pulse wave velocity (baPWV) in all four limbs^18,19^. In addition, all personnel involved in data collection were trained healthcare staff or research personnel who had received standardized instruction before data acquisition. Second, all participants were assessed under uniform data acquisition procedures; specifically, each participant was instructed to remain seated and at rest for approximately five minutes prior to the measurements.

All participants in this study were recruited from Hubei Province, China, and the findings may not be directly generalizable to populations in other regions or those with different ethnic or racial backgrounds. The age distribution of the participants was uneven, with a higher proportion of middle-aged and older adults, resulting in limited representation of younger individuals. Additionally, detailed medical histories (e.g., diabetes, cardiovascular diseases) were not collected, preventing further assessment of the potential impact of comorbidities on arterial stiffness characteristics or the performance of the VA prediction models.

### Experimental design

**Fig. 1:**
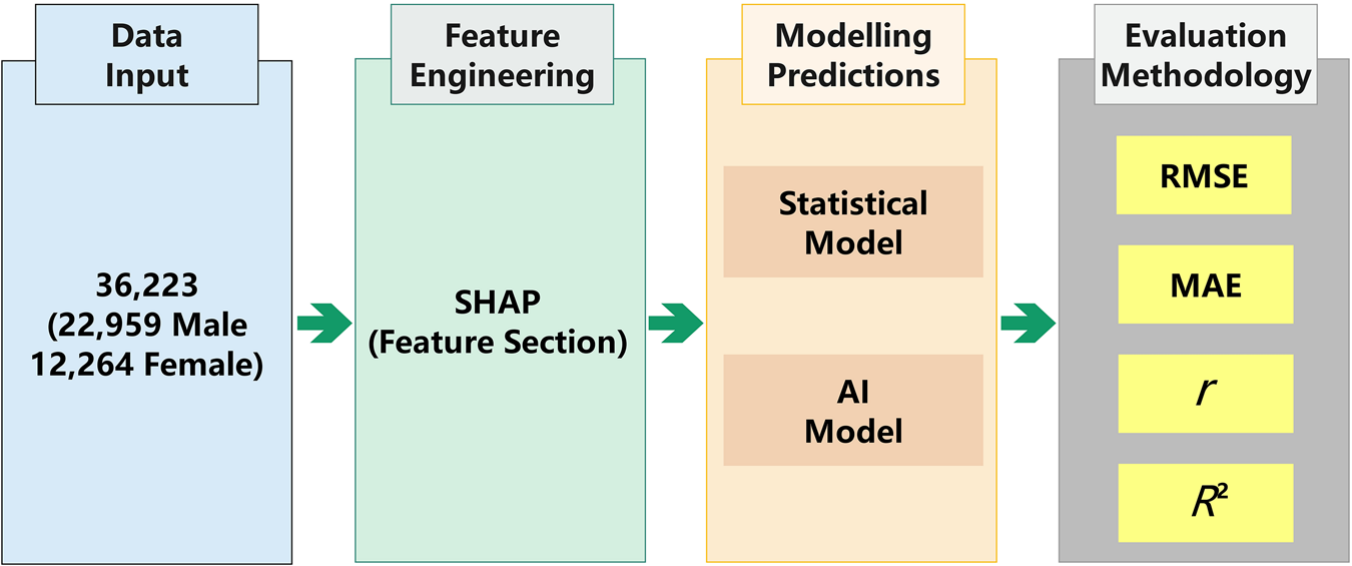
Workflow for vascular age prediction.

In this study, we evaluated seven predictive approaches, including one statistical model (KDM) and six AI models: MLR, LASSO regression, RF, SVR, XGBoost, and DNN. A SHAP-based strategy was adopted to support both feature selection and model interpretability. Features were screened within each repeated split using an XGBoost-based selector, and the retained predictors were then used as inputs to the seven models. SHAP analyses were further used to characterize feature contribution patterns at both the global and individual levels. Model performance was evaluated using root mean square error (RMSE), mean absolute error (MAE), Pearson correlation coefficient (*r*), and the coefficient of determination (*R*^2^). The complete workflow is illustrated in Fig. 1.

### Data splitting and preprocessing

The male and female cohorts were analyzed separately. For each sex-specific dataset, repeated random splitting was performed 20 times with different random seeds. In each repetition, the dataset was divided into a training set (70%), a validation set (15%), and a test set (15%). Specifically, the data were first split into training-plus-validation (85%) and test (15%) subsets, after which the training-plus-validation subset was further divided into training and validation subsets so that the final proportions were 70%, 15%, and 15%.

In the benchmark experiments, CA was used as the supervised regression target. The resulting model-derived age estimate was used as a proxy for VA, under the assumption that arterial stiffness biomarkers capture biological aging of the vasculature. Input features were derived from the arterial stiffness dataset after excluding the variables sex and label. All feature columns were converted to numeric format. Columns containing only missing values or constant values were removed. For data curation and downstream benchmarking, remaining missing values were filled using the median of the corresponding feature within each sex-specific dataset.

Feature selection was performed separately within each repetition using SHAP values. An XGBoost regressor was first trained on the training subset, and the mean absolute SHAP value of each feature was computed. Features with mean absolute SHAP values greater than 1.5 were retained. This cutoff was chosen empirically as a heuristic criterion to preserve the most influential vascular features while avoiding an unnecessarily large input set. Because SHAP magnitudes may vary across repeated splits, the threshold was used as a practical screening rule rather than a strict biological or statistical boundary. If fewer than five features met this criterion, the top five ranked features were selected instead. The selected threshold mainly served to standardize feature screening across repeated splits and to facilitate benchmarking under a consistent modelling workflow. This procedure yielded a compact set of informative vascular features for downstream modelling.

For MLR, LASSO, SVR, RF, and XGBoost, feature standardization was implemented through a scikit-learn pipeline, and hyperparameters were optimized by five-fold cross-validation on the training subset only. For the DNN model, features were standardized using a scaler fitted on the training subset and then applied to the validation and test subsets. Early stopping based on validation RMSE was used to prevent overfitting. The KDM model was fitted using the selected training features and subsequently evaluated on the held-out test subset within each repetition.

### Modelling predictions

The SHAP-selected arterial stiffness features were subsequently input into seven predictive approaches for VA estimation, including one statistical model (KDM) and six AI models: MLR, LASSO regression, RF, SVR, XGBoost, and DNN. These complementary approaches enabled modelling of age-related vascular characteristics from both statistical and machine learning perspectives, while also supporting benchmarking of the curated arterial stiffness dataset under repeated data splits.

The KDM is a classical statistical model adopted in this study. Originally developed by Klemera and Doubal^20^, this approach estimates biological age from multiple biomarkers by combining feature-wise regressions with an overall age-regression adjustment^20^. In the present study, the KDM framework was applied using the SHAP-selected arterial stiffness variables available in the dataset. The fundamental algorithmic principle can be mathematically expressed as:

1 $$\,{\rm{VA}}\,=\frac{{\sum }_{j=1}^{m}\left({x}_{j}-{g}_{j}\right)\frac{{h}_{j}}{{s}_{j}^{2}}+\frac{CA}{{s}_{D}^{2}}}{{\sum }_{j=1}^{m}{\left(\frac{{h}_{j}}{{s}_{j}}\right)}^{2}+\frac{1}{{s}_{D}^{2}}}$$

where, in the KDM formula, *m* denotes the total number of extracted features. The CA variable refers to the participant’s chronological age. *x*_*j*_ corresponds to the observed value of the *j*^*th*^ feature. *g*_*j*_ represents the intercept from the regression of the *j*^*th*^ feature on VA; however, since VA cannot be directly measured, CA is used as a proxy. *h*_*j*_ is the regression slope of the *j*^*th*^ feature with respect to CA. *s*_*j*_ indicates the root mean square error (RMSE) obtained from this regression. Finally, ${s}_{D}^{2}$ captures the overall RMSE when regressing all processed features on CA, serving as an adjustment for the unobservable VA^20,21^.

The MLR model was used as a baseline learning approach for predicting VA from arterial stiffness data. This model establishes a linear relationship between VA and multiple predictors derived from the dataset, including baPWV, blood pressure-related parameters, ABI, and other selected vascular measurements. Because the male and female cohorts were modeled separately, sex was not included as an explanatory variable within the sex-specific models. Although less flexible than nonlinear learning approaches, MLR provides interpretable coefficients and serves as a useful benchmark for model comparison^22^. The regression formulation follows this structure:

2 $$\,{\rm{VA}}\,={\beta }_{0}+{\beta }_{1}{x}_{1}+{\beta }_{2}{x}_{2}+\cdots +{\beta }_{p}{x}_{p}+\epsilon $$

Within the framework of the MLR model, *β*_0_ represents the intercept, while *β*_1_, *β*_2_, …, *β*_*p*_ quantify the contribution of the explanatory variables *x*_1_, *x*_2_, …, *x*_*p*_ to the predicted VA. In this study, the explanatory variables correspond to the input features derived from the arterial stiffness dataset, including measurements such as baPWV, blood pressure indices, ABI, and related hemodynamic parameters. The residual term *ϵ* represents the unexplained variation in the model.

LASSO regression was applied to predict VA using arterial stiffness data. By imposing an *L*_1_ − *norm* penalty, LASSO performs variable selection and regularization, effectively handling multicollinearity while enhancing model interpretability^23^. Its simplicity and parsimony make it a practical benchmark for VA estimation.

RF, an ensemble learning method, was implemented for VA prediction. By aggregating multiple decision trees, RF captures complex nonlinear interactions within the data and provides variable importance measures, offering insights into factors contributing to vascular aging^24^.

SVR was employed to explore nonlinear relationships in the dataset. Through kernel functions, SVR captures complex patterns while maintaining strong generalization capability, making it well-suited for high-dimensional feature spaces.

XGBoost, based on gradient-boosted decision trees^25^, was used to enhance prediction. Its ability to model intricate data patterns with built-in regularization and high computational efficiency makes it particularly suitable for large-scale biomedical datasets^26^.

DNN leveraged multiple nonlinear layers to learn hierarchical representations from the data. This architecture captures complex interactions and extracts high-level features beyond the capacity of traditional models, with performance improving as data volume and optimization increase.

### Evaluation methodology

Root Mean Square Error (RMSE)

Measures the standard deviation of prediction errors:

3 $$\,{\rm{RMSE}}\,=\sqrt{\frac{1}{n}{\sum }_{i=1}^{n}{({y}_{i}-{\widehat{y}}_{i})}^{2}}$$

Mean Absolute Error (MAE)

Computes the average absolute error magnitude:

4 $$\,{\rm{MAE}}\,=\frac{1}{n}\mathop{\sum }\limits_{i=1}^{n}| {y}_{i}-{\widehat{y}}_{i}| $$

Correlation Coefficient (*r*)

Quantifies linear dependence between predictions and observations:

5 $$r=\frac{{\sum }_{i=1}^{n}({y}_{i}-\bar{y})({\widehat{y}}_{i}-\bar{\widehat{y}})}{\sqrt{{\sum }_{i=1}^{n}{({y}_{i}-\bar{y})}^{2}{\sum }_{i=1}^{n}{({\widehat{y}}_{i}-\bar{\widehat{y}})}^{2}}}$$

R-squared(*R*^2^)

Represents the proportion of explained variance:

6 $${R}^{2}=1-\frac{{\sum }_{i=1}^{n}{({y}_{i}-{\widehat{y}}_{i})}^{2}}{{\sum }_{i=1}^{n}{({y}_{i}-\bar{y})}^{2}}$$

where *y*_*i*_ denotes chronological age, ${\widehat{y}}_{i}$ denotes the model-predicted age (used here as a proxy for vascular age), $\bar{y}$ is the mean chronological age, $\bar{\widehat{y}}$ is the mean predicted vascular age, and *n* is the sample size.

### De-identification

During dataset creation, all direct personal identifiers were removed. Only demographic variables required for analysis (sex and age) and arterial stiffness measurements were retained in the released dataset.

## Data Records

The dataset is provided as a single Microsoft Excel (*.xlsx) file, with each row corresponding to one participant^27^. The file contains demographic variables (sex, age), vascular stiffness parameters (e.g., baPWV, ABI, blood pressure indices), and a binary label indicating vascular aging status. All personal identifiers have been completely removed.

## Technical Validation

### Model training and validation

Model development and evaluation followed the repeated random-split workflow described in the Methods section. Briefly, the male and female cohorts were analyzed separately over 20 repetitions, with feature selection performed within each repetition and model performance evaluated on the corresponding held-out test subset. Performance was summarized using MAE, RMSE, *r*, and *R*^2^, and bootstrap resampling was used to estimate 95% confidence intervals for the average metric values across repetitions.

### Statistical comparison of model performance

Overall differences among the six AI models (MLR, LASSO, RF, SVR, XGBoost, and DNN) were assessed using the Friedman test on repetition-level MAE and RMSE values across the 20 repeated random splits Table 2. Bootstrap resampling (5,000 iterations) was additionally used to estimate 95% confidence intervals for the average performance metrics of each model, with the detailed results summarized in Table 3, confirming the stability of the benchmark results across repeated data splits. Across both male and female cohorts, KDM yielded the lowest error values under the current benchmark setting, while the nonlinear learning-based models generally outperformed the linear baselines. Among the learning-based approaches, SVR and XGBoost showed comparatively strong performance Table 4.

**Table 2:** Friedman test results for overall model differences across 20 repeated random splits.

Sex	Metric	Models compared	Friedman *χ*^2^	*P* value
Male	MAE	KDM, MLR, LASSO, RF, SVR, XGBoost, DNN	115.05	<0.001
Male	RMSE	KDM, MLR, LASSO, RF, SVR, XGBoost, DNN	104.40	<0.001
Female	MAE	KDM, MLR, LASSO, RF, SVR, XGBoost, DNN	110.85	<0.001
Female	RMSE	KDM, MLR, LASSO, RF, SVR, XGBoost, DNN	105.02	<0.001

Friedman tests were conducted on repetition-level MAE and RMSE values across 20 repeated random splits.

**Table 3:** Bootstrap summary of model performance across repeated splits.

Sex	Model	MAE (95% CI)	RMSE (95% CI)	*r* (95% CI)	*R*^2^ (95% CI)
Male	KDM	6.53 (6.48–6.58)	8.56 (8.47–8.64)	0.822 (0.820–0.824)	0.498 (0.488–0.509)
Male	SVR	7.09 (7.06–7.12)	8.91 (8.87–8.94)	0.678 (0.676–0.681)	0.457 (0.453–0.461)
Male	XGBoost	7.15 (7.12–7.18)	8.89 (8.85–8.92)	0.678 (0.675–0.681)	0.459 (0.455–0.463)
Male	DNN	7.17 (7.13–7.21)	8.92 (8.88–8.97)	0.678 (0.675–0.681)	0.455 (0.450–0.460)
Male	RF	7.18 (7.15–7.21)	8.93 (8.90–8.96)	0.674 (0.671–0.676)	0.453 (0.450–0.457)
Male	MLR	7.58 (7.54–7.61)	9.39 (9.35–9.43)	0.630 (0.627–0.633)	0.396 (0.392–0.400)
Male	LASSO	7.58 (7.54–7.61)	9.39 (9.35–9.43)	0.630 (0.627–0.633)	0.396 (0.392–0.400)
Female	KDM	5.48 (5.44–5.52)	7.12 (7.05–7.19)	0.862 (0.859–0.865)	0.634 (0.625–0.643)
Female	SVR	6.03 (5.98–6.08)	7.65 (7.59–7.72)	0.761 (0.755–0.766)	0.578 (0.569–0.586)
Female	XGBoost	6.07 (6.01–6.12)	7.65 (7.58–7.71)	0.761 (0.755–0.766)	0.578 (0.569–0.587)
Female	RF	6.10 (6.05–6.16)	7.70 (7.63–7.76)	0.757 (0.751–0.763)	0.573 (0.564–0.581)
Female	DNN	6.11 (6.06–6.16)	7.69 (7.63–7.75)	0.761 (0.756–0.767)	0.573 (0.565–0.581)
Female	MLR	6.37 (6.32–6.42)	8.01 (7.94–8.07)	0.734 (0.728–0.739)	0.538 (0.529–0.546)
Female	LASSO	6.37 (6.32–6.42)	8.01 (7.94–8.07)	0.734 (0.728–0.739)	0.538 (0.529–0.546)

**Table 4:** Cross-model global SHAP ranking summary in the male and female cohorts.

Sex	Feature	Mean rank	Mean absolute SHAP	Top-1 frequency
Male	LbaPWV	1.63	3.256	69.2%
Male	LbPMap	1.98	2.409	4.2%
Male	RbaPWV	3.00	1.835	29.6%
Male	RbPMap	3.37	1.587	0.0%
Male	LaMAP	4.83	0.403	0.0%
Male	RaMAP	4.85	0.994	0.0%
Female	LbaPWV	1.01	3.425	99.2%
Female	LbPMap	2.21	2.349	0.0%
Female	RbPMap	2.78	2.118	0.8%
Female	RbaPWV	4.01	1.221	0.0%
Female	LaMAP	4.99	0.628	0.0%
Female	RaMAP	5.00	0.594	0.0%

Mean rank, mean absolute SHAP value, and top-1 frequency were summarized across all model–repeat combinations within each sex-specific cohort.

**Fig. 2:**
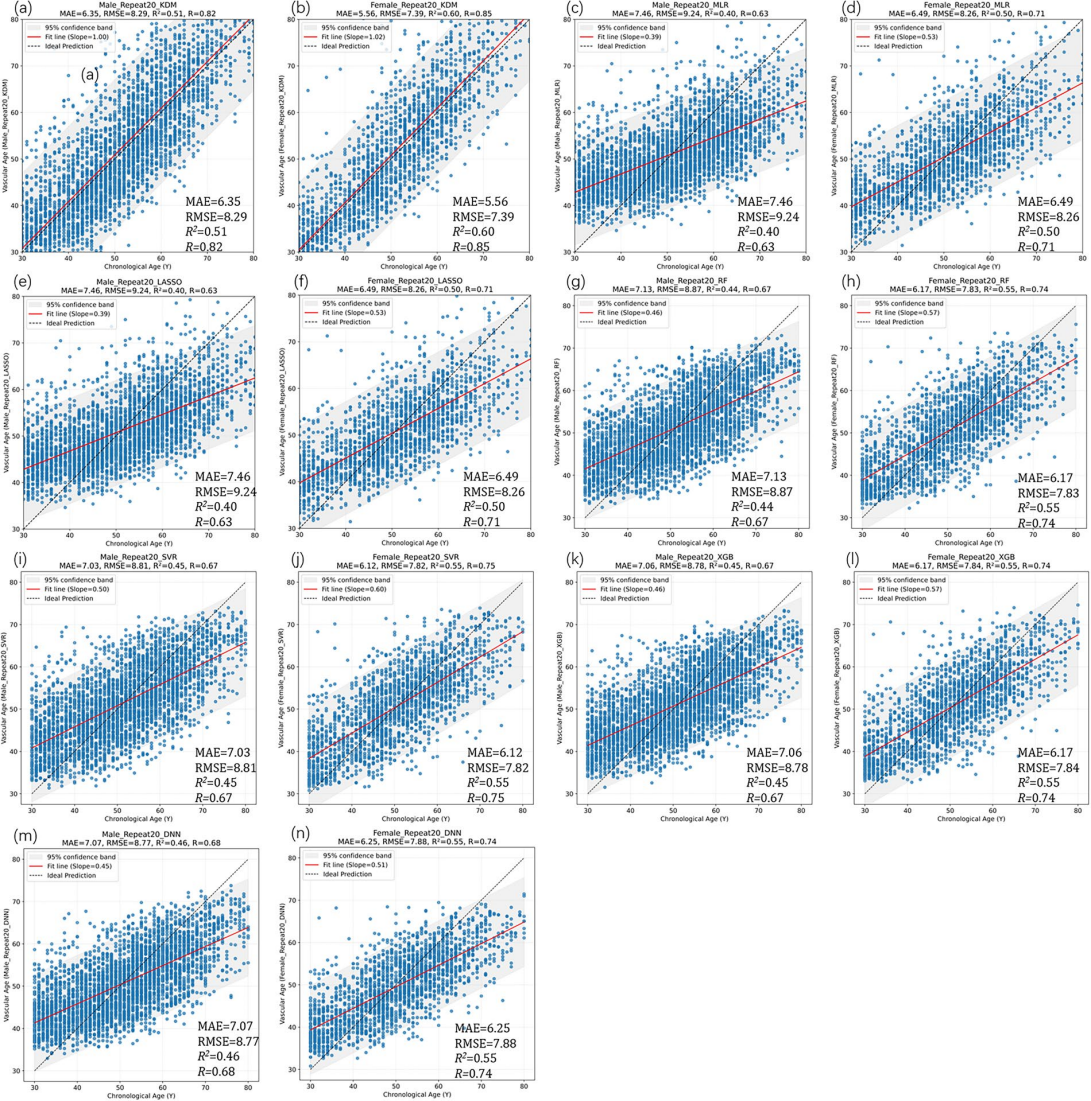
Scatter plots of predicted vascular age versus chronological age across models in male and female cohorts. Panels (**a**)–(**n**) show prediction results stratified by sex, with each pair of subplots corresponding to a specific model (male on the left and female on the right). Specifically, (**a**,**b**) KDM, (**c**,**d**) MLR, (**e**,**f**) LASSO, (**g**,**h**) RF, (**i**,**j**) SVR, (**k**,**l**) XGBoost, and (**m**,**n**) DNN. Each scatter plot illustrates the relationship between predicted vascular age (VA) and chronological age (CA). The dashed diagonal line represents the line of identity (VA = CA), while the red line indicates the fitted regression line. Shaded regions denote the 95% confidence intervals. Model performance metrics, including MAE, RMSE, Pearson’s correlation coefficient (*r*), and coefficient of determination (*R*^2^), are reported within each subplot.

**Fig. 3:**
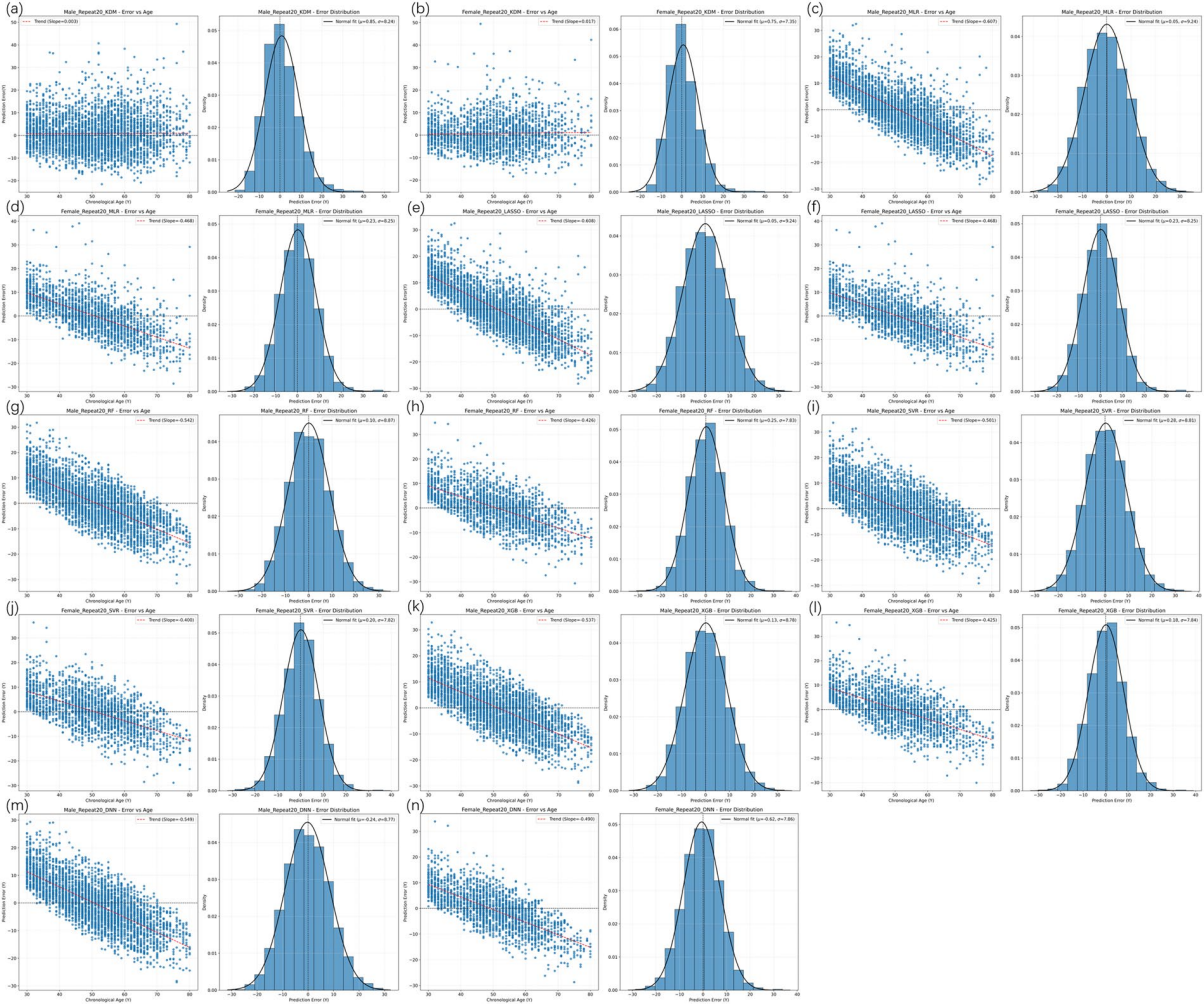
Residual trends and error distributions of predictive models in male and female cohorts. Each pair of panels corresponds to a specific model (KDM, MLR, LASSO, RF, SVR, XGBoost, and DNN), with the left panel showing residuals versus chronological age (CA) and the right panel displaying the corresponding error distribution. The residual plots illustrate age-related patterns in prediction errors, while the histograms, overlaid with fitted normal distribution curves, characterize the overall error distributions. Compared with linear baseline models, the better-performing nonlinear approaches generally exhibit a narrower spread of errors and more symmetric residual distributions, indicating improved stability and robustness across repeated data splits.

To better visualize these findings, prediction scatter plots and error distribution plots are presented in Figs. 2 and 3, respectively Table 5.

**Table 5:** Cross-model global SHAP feature importance in male and female cohorts, summarized as mean absolute SHAP values across 20 repeated data splits.

Model	Male			Female		
	Feature	Mean absolute SHAP value	Rank	Feature	Mean absolute SHAP value	Rank
MLR	RbaPWV	2.670 ± 0.284	1	LbaPWV	3.110 ± 0.263	1
	LbPMap	2.267 ± 0.138	2	LbPMap	2.236 ± 0.100	2
	LbaPWV	1.805 ± 0.636	3	RbPMap	1.945 ± 0.134	3
	RbPMap	1.324 ± 0.104	4	RbaPWV	1.197 ± 0.159	4
	RaMAP	0.930 ± 0.154	5	LaMAP	0.394 ± 0.057	5
	LaMAP	0.143 ± 0.160	6	RaMAP	0.267 ± 0.027	6
LASSO	RbaPWV	2.667 ± 0.284	1	LbaPWV	3.110 ± 0.263	1
	LbPMap	2.266 ± 0.138	2	LbPMap	2.236 ± 0.100	2
	LbaPWV	1.803 ± 0.633	3	RbPMap	1.945 ± 0.134	3
	RbPMap	1.323 ± 0.104	4	RbaPWV	1.194 ± 0.159	4
	RaMAP	0.923 ± 0.160	5	LaMAP	0.391 ± 0.057	5
	LaMAP	0.123 ± 0.174	6	RaMAP	0.264 ± 0.027	6
SVR	LbaPWV	3.722 ± 0.455	1	LbaPWV	2.829 ± 0.207	1
	LbPMap	2.395 ± 0.183	2	LbPMap	2.366 ± 0.154	2
	RbPMap	1.990 ± 0.173	3	RbPMap	2.189 ± 0.178	3
	RbaPWV	1.607 ± 0.202	4	RbaPWV	1.635 ± 0.159	4
	RaMAP	1.037 ± 0.154	5	LaMAP	0.787 ± 0.068	5
	LaMAP	0.685 ± 0.061	6	RaMAP	0.767 ± 0.007	6
*RF	LbaPWV	4.648 ± 0.259	1	LbaPWV	4.549 ± 0.300	1
	LbPMap	2.621 ± 0.148	2	LbPMap	2.370 ± 0.309	2
	RbPMap	1.537 ± 0.157	3	RbPMap	2.248 ± 0.225	3
	RbaPWV	0.932 ± 0.105	4	RbaPWV	0.774 ± 0.119	4
	RaMAP	0.872 ± 0.110	5	RaMAP	0.622 ± 0.016	5
	LaMAP	0.398 ± 0.018	6	LaMAP	0.590 ± 0.027	6
XGB	LbaPWV	4.173 ± 0.402	1	LbaPWV	3.720 ± 0.256	1
	LbPMap	2.419 ± 0.143	2	LbPMap	2.691 ± 0.124	2
	RbPMap	1.641 ± 0.119	3	RbPMap	2.148 ± 0.138	3
	RbaPWV	1.420 ± 0.142	4	RbaPWV	1.104 ± 0.164	4
	RaMAP	1.026 ± 0.145	5	RaMAP	0.753 ± 0.010	5
	LaMAP	0.547 ± 0.020	6	LaMAP	0.744 ± 0.047	6
DNN	LbaPWV	3.385 ± 0.599	1	LbaPWV	3.232 ± 0.386	1
	LbPMap	2.483 ± 0.286	2	RbPMap	2.235 ± 0.215	2
	RbaPWV	1.712 ± 0.263	3	LbPMap	2.197 ± 0.230	3
	RbPMap	1.708 ± 0.233	4	RbaPWV	1.423 ± 0.265	4
	RaMAP	1.174 ± 0.262	5	RaMAP	0.891 ± 0.057	5
	LaMAP	0.526 ± 0.179	6	LaMAP	0.865 ± 0.163	6

Values are presented as mean ± standard deviation of mean absolute SHAP values across 20 repeated random splits.

### Model interpretability analysis

To enhance interpretability, model-specific SHAP analyses were conducted for the fitted learning-based models. For tree-based models, TreeExplainer was used; for linear models, LinearExplainer was adopted; and for other models, KernelExplainer or DeepExplainer was used when appropriate. SHAP summary plots and bar plots were generated to visualize global feature importance and to compare ranking consistency across models and repeated splits. Local SHAP explanations were also examined at the individual-sample level to illustrate how specific features increased or decreased predicted vascular age in representative observations. Because the repeated-split analysis generated a large number of sample-level explanations, the main text primarily emphasizes global SHAP patterns, while representative local SHAP results are summarized in Table 6.

**Table 6:** Representative local SHAP features by sex and model.

Model	Direction	Male	Female
MLR	Increase VA	RaMAP 0.899 (0.241) LbPMap 2.460 (0.224) RbPMap 1.440 (0.187)	LaMAP 0.369 (0.225) LbPMap 2.343 (0.221) RbPMap 2.048 (0.211)
MLR	Decrease VA	RbaPWV -2.352 (0.218) LbPMap -2.291 (0.213) LbaPWV -1.634 (0.211)	LbaPWV -2.735 (0.265) LbPMap -2.404 (0.203) RbPMap -2.140 (0.196)
LASSO	Increase VA	RaMAP 0.893 (0.241) LbPMap 2.459 (0.226) RbPMap 1.437 (0.189)	LaMAP 0.366 (0.225) LbPMap 2.342 (0.221) RbPMap 2.048 (0.211)
LASSO	Decrease VA	RbaPWV -2.349 (0.220) LbPMap -2.290 (0.214) LbaPWV -1.630 (0.213)	LbaPWV -2.735 (0.265) LbPMap -2.403 (0.203) RbPMap -2.140 (0.196)
RF	Increase VA	RaMAP 0.816 (0.284) LbPMap 2.268 (0.262) RbPMap 1.093 (0.221)	RbPMap 1.762 (0.270) LaMAP 0.524 (0.244) LbPMap 2.183 (0.225)
RF	Decrease VA	LbaPWV -3.897 (0.287) RbaPWV -0.777 (0.260) LbPMap -3.368 (0.172)	LbaPWV -3.644 (0.310) RbaPWV -0.769 (0.245) LbPMap -2.808 (0.211)
SVR	Increase VA	RbPMap 1.649 (0.243)RaMAP 0.855 (0.240)LbPMap 2.034 (0.235)	RbPMap 1.662 (0.263)LbPMap 2.160 (0.242)LaMAP 0.663 (0.197)
SVR	Decrease VA	LbaPWV -3.501 (0.259)RbaPWV -1.219 (0.235)LbPMap -3.186 (0.184)	LbaPWV -2.590 (0.271)RbaPWV -1.205 (0.255)LbPMap -2.952 (0.202)
XGBoost	Increase VA	RaMAP 0.848 (0.278)LbPMap 2.207 (0.239)RbPMap 1.289 (0.223)	RbPMap 1.816 (0.255)LaMAP 0.519 (0.245)LbPMap 2.451 (0.238)
XGBoost	Decrease VA	LbaPWV -3.683 (0.278)RbaPWV -1.158 (0.243)LbPMap -2.909 (0.190)	LbaPWV -3.225 (0.284)RbaPWV -0.915 (0.262)LbPMap -3.155 (0.205)
DNN	Increase VA	RaMAP 0.950 (0.254)RbPMap 1.379 (0.229)LbPMap 2.212 (0.229)	LaMAP 0.641 (0.247)RbPMap 1.740 (0.247)LbPMap 2.029 (0.211)
DNN	Decrease VA	LbaPWV -3.135 (0.260)RbaPWV -1.371 (0.230)LbPMap -3.105 (0.194)	LbaPWV -2.890 (0.279)RbaPWV -1.213 (0.224)LbPMap -2.653 (0.205)

For each sex-specific model, the top three features increasing predicted vascular age (VA) and the top three features decreasing predicted VA are shown. Values are presented as *mean SHAP* (frequency).

## Usage Notes

The dataset contains a comprehensive record of arterial stiffness measurements and demographic information for 36,223 participants. A summary of all variables is provided in Table 7. Each entry includes demographic data, hemodynamic parameters, and a label variable indicating vascular aging status^28^.

**Table 7:** Description of variables in the arterial stiffness dataset.

Variable	Description
Sex	Sex of the participant
Age	Chronological age of the participant (years)
LbaPWV	Left brachial-ankle pulse wave velocity (m/s), indicator of arterial stiffness
RbaPWV	Right brachial-ankle pulse wave velocity (m/s), indicator of arterial stiffness
LbPMap	Left brachial mean arterial pressure (mmHg)
RbPMap	Right brachial mean arterial pressure (mmHg)
LaSYS	Left ankle systolic pressure (mmHg)
RaSYS	Right ankle systolic pressure (mmHg)
LaMAP	Left ankle mean arterial pressure (mmHg)
RaMAP	Right ankle mean arterial pressure (mmHg)
Labi	Left ankle-brachial index (ABI)
Rabi	Right ankle-brachial index (ABI)
Label	Vascular aging status

Sex was coded as 0 for female and 1 for male. Label denotes a derived binary grouping variable included in the released dataset. Specifically, participants with RbaPWV 15.2 m/s, LbaPWV ≤ 15.2 m/s, and RaSYS < 140 mmHg were assigned a label of 0, whereas all remaining participants were assigned a label of 1. In the present regression analyses, Label and Sex were excluded from the predictor set before model training.

The data are stored in a Microsoft Excel (*.xlsx) file, ensuring compatibility with widely used analysis platforms such as Python, R, MATLAB, and SPSS. Clear column names, as described in Table 7, facilitate straightforward data import and preprocessing.

This structured dataset can be applied to a wide range of analyses, including VA modelling, arterial stiffness assessment, and cardiovascular risk stratification. Prior to modelling, it is recommended to perform data cleaning, such as outlier detection and normalization of continuous variables, particularly when using machine learning methods like SVR, RF, or DNN. The standardized format supports reproducible workflows and scalable analysis pipelines.

## Supplementary information

Ethical Permission

## Data Availability

The arterial stiffness dataset is openly available on Figshare^27^. The dataset can be directly imported into widely used platforms such as Python, R, MATLAB, and SPSS. Source code and implementation notes are archived at GitHub: https://github.com/dpc168/A-curated-arterial-stiffness-dataset-for-vascular-age-prediction-in-China.

## Code availability

We implemented all procedures using Python version 3.9. To guarantee reproducibility and enable future research extensions, both the source code and implementation notes are publicly archived at GitHub (https://github.com/dpc168/A-short-recorded-arteriosclerosis-dataset-for1-vascular-age-prediction-in-China).
