# Supplementary material for: A curated arterial stiffness dataset for vascular age prediction in China
Source: Sci Data. 2026 Apr 28;13:967. doi: 10.1038/s41597-026-07276-2 (PMC13332198; doi:10.1038/s41597-026-07276-2)
Supplement: Supplementary file 1 — Ethical Permission [file 41597_2026_7276_MOESM1_ESM.pdf]

Exemption From Ethical Review Report

**Review Agency:**	Academic Ethics Committee of Anqing Normal University	**No:**	AQNU2025039
**Title:**	Both chronological age and individual differences in aging are the two indispensable components for predicting biological age		
**Principal Investigator:**	Qingfeng TANG	**E-mail:**	tqf0913@aqnu.edu.cn
**Principal Co-Investigators:**	Hui AN (Xiangyang Central Hospital, anvui@126.com);		
**Study Dates:**	2025.1.1-2030.1.1		
**Exemption Reason:**	This study uses anonymous/de identified biomedical data.		
**Data Source:**	The data used in this study comes from Xiangyang Central Hospital. All data has been de identified and does not contain sensitive information. All data cannot be associated with individuals.		
**Study contents:**	This study uses artificial intelligence methods to analyze arteriosclerosis indices and predict vascular biological age. The research content does not involve new data collection or patient intervention.		
**Compliance Statements:**	This study complies with the exemption criteria of the Academic Ethics Committee of Anqing Normal University and the Helsinki Declaration.		
**Exemption Decision:**	☒ Exempt	☐Not Exempt	
**Seal for Review Agency:**	Academic Ethics Committee of Anqing Normal University<br>May. 1, 2025<br>安庆师范大学 学术委员会科学研究与学术道德（科学伦理）专门委员会		
